# The role of the medial frontal cortex in the maintenance of emotional states

**DOI:** 10.1093/scan/nsu011

**Published:** 2014-03-10

**Authors:** Christian E. Waugh, Maria G. Lemus, Ian H. Gotlib

**Affiliations:** ^1^Wake Forest University, P.O. Box 7778, Winston-Salem, NC 27109, USA and ^2^Stanford University, 450 Serra Mall, Stanford, CA 94305

**Keywords:** emotion, working memory, medial frontal cortex, affect maintenance, emotion regulation

## Abstract

Evidence is accruing that people can maintain their emotional states, but how they do it and which brain regions are responsible still remains unclear. We examined whether people maintain emotional states ‘actively’, with explicit elaboration of the emotion, or ‘passively’, without elaboration. Twenty-four participants completed an emotion maintenance task in which they either maintained the emotional intensity from the first picture of a pair to compare to that of the second picture (‘maintain’ condition), or only rated their emotional response to the second picture (‘non-maintain’ condition). Supporting the ‘active’ maintenance hypothesis, when maintaining *vs* not maintaining emotion, participants exhibited increased height and width of activation in the dorsal medial frontal cortex (MFC) and lateral prefrontal cortex, regions associated with explicit emotion generation and manipulation of contents in working memory, respectively. Supporting the ‘passive’ maintenance hypothesis, however, when viewing negative emotional pictures (*vs* neutral pictures) that were not explicitly maintained, participants exhibited greater duration of activity in the rostral MFC, a region associated with implicit emotion generation. Supported by behavioral findings, this evidence that people maintain emotional states both naturally in the rMFC and strategically in the dMFC may be critical for understanding normal as well as disordered emotion regulation.

Maintaining an emotional state is a fundamental aspect of emotional life, and one of the three primary forms of emotion regulation (in addition to up- and down-regulation; Parrott, 1993; Gross, 1998). For example, intentionally maintaining an emotional state can enhance performance on subsequent emotion-congruent tasks (Tamir *et al.*, 2008). Emotion maintenance is also a form of emotion dysregulation in psychopathology. Individuals diagnosed with depression, for example, are characterized by the persistent maintenance of negative emotional states (Gilboa and Gotlib, 1997), negative thoughts (Nolen-Hoeksema and Morrow, 1993) and stress-induced physiology (Young *et al.*, 2000). In contrast, people diagnosed with schizophrenia show deficits in maintaining negative and positive emotion (Gard *et al.*, 2011).

Researchers have often operationalized emotion maintenance as the cognitive maintenance of emotionally valenced information, such as emotionally charged words (Perlstein *et al.*, 2002) and images (Levens and Gotlib, 2010). In those studies, investigators typically substitute emotional stimuli for non-emotional stimuli in standard working-memory tasks to examine changes in people’s maintenance of verbal/pictoral stimuli as a function of the emotionality of the stimuli. More relevant to the aims of this study, however, recent studies have shown that people can also maintain the emotional states induced by emotional stimuli as a source of information (Mikels *et al.*, 2008). In a modified delayed match-to-sample task, Mikels *et al.* (2008) demonstrated that people can hold in mind their emotional response to an image to compare it to their emotional response to a subsequent, different image. Mikels *et al.* also showed that emotional state working memory involves at least a partially different system than does cognitive working memory—intervening cognitive distracters did not interfere with the maintenance of emotional states.

In a follow-up to the Mikels *et al.* (2008) study, investigators examined possible mechanisms that underlay emotion maintenance (C.E. Waugh and I.H. Gotlib, unpublished data). These investigators adapted Mikels *et al.*’s emotional working memory task, but instead of using a cognitive working memory task as the control condition, devised a ‘non-maintenance’ of emotion condition to separate the effects of maintaining emotion from the effects of viewing emotional stimuli more generally. Waugh and Gotlib found that participants maintained their emotional facial expressions during the delay period between pictures on maintenance trials and that this activity predicted which of the two pictures evoked a more intense emotional response. This finding advanced ‘embodiment of emotion’ models that posit that afferent information from the body can serve as information about one’s emotional state (Niedenthal, 2007) by showing that this bodily information can be strategically maintained past the end of the stimulus.

In this study, we tested two competing, but not mutually exclusive, hypotheses concerning how people strategically maintain emotional states. The ‘passive maintenance’ hypothesis is that people maintain their emotional states by maintaining the initially generated emotional responses to that stimulus in their original form. In this case, maintaining an emotional state would resemble the passive maintenance of information in simple delayed match-to-sample tasks, which does not necessarily require additional manipulation (D'esposito *et al.*, 1999). If this hypothesis is correct, then we would expect to find greater activation during maintenance of emotion than during non-maintenance of emotion in brain regions associated with the primary/initial generation of emotional responses, such as the rostral medial frontal cortex (rMFC), amygdala and/or insula.

The rMFC is involved with the ‘meaning-making’ of emotional stimuli—generating the primary appraisals of the salience and relevance of emotional stimuli to the self (Roy *et al.*, 2012), and the corresponding physiological responses (Wager *et al.*, 2009). Consistent with its currently hypothesized role in the maintenance of emotion, the rMFC exhibits prolonged duration of activity in response to emotional events (Waugh *et al.*, 2010), which tracks the duration of the emotional event (Wager *et al.*, 2009), especially for people who are susceptible to experiencing sustained negative emotional responses (Waugh *et al.*, 2012). The amygdala and insula are also associated with the initial reactivity to an emotional stimulus (especially negative; see Lindquist *et al.*, 2012 for review) and have been shown to exhibit prolonged duration of activation in enduring emotional situations (Herry *et al.*, 2007; Waugh *et al.*, 2010).

C.E. Waugh and I.H. Gotlib (unpublished data) also found that maintaining emotional states led to an increase in the recalled emotional intensity of the images relative to when participants did not have to maintain their emotional states. This finding prompts an alternative, ‘active maintenance’ hypothesis that people do not maintain their emotional states via a passive maintenance of initial emotional responses, but rather, by intentionally elaborating on their emotional responses. In this case, maintaining an emotional state would resemble the active maintenance of information in working memory reflected by processes such as rehearsal (Smith and Jonides, 1998) or manipulation (D'sposito *et al.*, 1999). This alternative hypothesis predicts greater activation during the maintenance of emotion than during the non-maintenance of emotion in brain regions associated with both the up-regulation of emotion, such as the dorsal MFC (dMFC), and the general executive functioning related to actively maintaining working memory content, such as the dorsolateral prefrontal cortex (dlPFC).

Typically thought of as part of the ‘mentalizing network’ (Amodio and Frith, 2006), the dMFC has also been shown to be involved with the up-regulation of emotion (Ochsner *et al.*, 2004), in part because of its role in the intentional/conscious generation of emotional appraisals (Mechias *et al.*, 2010). The dMFC is activated more strongly when people view supraliminal, compared with subliminal, fear expressions (Williams *et al.*, 2006) and when people generate top-down appraisals of threatening stimuli in ‘instructed fear’ conditioning paradigms (Mechias *et al.*, 2010); it is also a significant part of the system responsible for intentionally generating and controlling physiological arousal (Critchley *et al.*, 2002). Preliminary evidence also suggests that the dMFC can exhibit sustained activation during emotional processing (Haas *et al.*, 2008), a critical aspect of emotion maintenance. According to the ‘active maintenance’ hypothesis, the dlPFC acts as the central executive (Smith and Jonides, 1999) that helps people actively maintain (*vs* passively maintain; D'esposito *et al.*, 1999) the emotional state generated by the dMFC.

In this study, we used the emotional state maintenance task that C.E. Waugh and I.H. Gotlib (unpublished data) adapted from Mikels *et al.* (2008) to examine the neural correlates of emotion maintenance. We tested two hypotheses. We predicted that if people ‘simply maintain’ their emotional states, relative to not maintaining emotional states, participants would exhibit greater activation in the rMFC, amygdala and/or insula when maintaining emotional states. Alternatively, we predicted that if people ‘actively maintain’ their emotional states, participants would exhibit greater activation in the dMFC and dlPFC when maintaining emotional states relative to not maintaining emotional states. Importantly, these two hypotheses predict different, but not mutually exclusive, patterns of activation. Indeed, this study is not designed to pit these two hypotheses against each other; rather, we present these alternative hypotheses as an a priori way of organizing expected patterns of activation. To further aid in identifying patterns of activation that are consistent with each of these hypotheses, we estimated the height and width of blood oxygenation-level dependent (BOLD) activation by using flexible time-variant hemodynamic response functions (HRFs) (Lindquist and Wager, 2007). This allowed us to assess whether activation in the targeted regions follows a ‘sustained’ pattern (i.e. greater width) that is consistent with the hypothesized maintenance processes.

## METHODS

### Participants

Twenty-seven right-handed females between the ages of 18 and 50 years (mean = 23.8 years, s.d. = 6.18) were recruited through internet community postings and flyers posted around Stanford University’s campus. To be eligible, participants had to have no history of head trauma, severe learning disabilities or physical limitations that prevented them from entering the magnetic resonance imaging (MRI) magnet. Informed consent was obtained from all participants, and all procedures were in accordance with the ethical standards as outlined by the Stanford Institutional Review Board. Participants were paid $25/h for their participation in the study. Three participants were excluded from data analyses due to errors in the programming software (two) or non-compliance to task instructions (one), leaving 24 participants.

### Emotional maintenance task

**Fig. 1:**
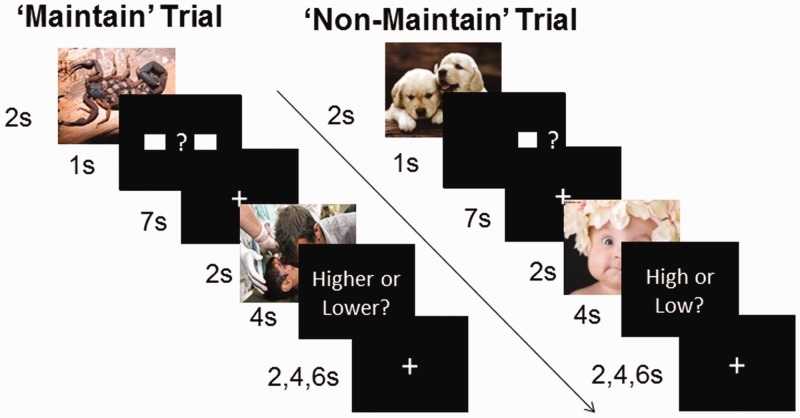
Emotion maintenance task. After viewing the first image of each pair, participants saw either two boxes signifying that they needed to maintain the emotional state from the first image to compare to that of the second image (‘maintain’ trials) or one box signifying that they were to rate their emotional response to only the second image (‘non-maintain’ trials).

Each trial consisted of five parts: first picture (2 s); one of two instruction slides (1 s); a black screen that served either as a waiting or retention interval (7 s); second picture (2 s); a screen prompting the participant to enter his/her response (4 s) and an inter-trial interval that was jittered among 2, 4 and 6 s (Figure 1). Pictures taken from the International Affective Picture System (Lang *et al.*, 1997) were selected to be positive, negative or neutral.

For each positive and negative emotion trial, participants were given one of two instructions after viewing the first picture. In the ‘non-maintain’ condition, participants viewed an instruction slide with one square and a question mark to its right, indicating that their task was to rate the intensity of the second picture. They did not rate the first picture, although they were not explicitly instructed to forget it. During the rating slide, participants reported the intensity of their emotional response to that second picture as ‘low’ (1) or ‘high’ (2). In the ‘maintain’ condition, participants viewed an instruction slide with two squares on either side of a question mark, indicating that their task was to compare the intensity of their emotional response to the first picture to the intensity of their emotional response to the second picture. During the rating slide, participants reported the intensity of their emotional response as being ‘lower’ (1) or ‘higher’ (2) during the second picture than during the first picture. For neutral trials, participants received only the ‘Non-maintain’ instructions (Figure 1).

Participants received 20 trials of each type: maintain-negative, maintain-positive, non-maintain-negative, non-maintain-positive and non-maintain-neutral, for a total of 100 trials separated into four blocks. To effectively separate the activation due to the first picture from that due to the second picture (Goghari and MacDonald, 2008), there were 20 catch trials (four for each trial type) in which participants did not see the second picture and did not make any ratings, but instead saw a gray screen. For half of the emotion trials, the first picture was normed as being more emotionally intense than the second picture, and *vice versa* for the other half of the emotion trials (see Supplementary data for more details about the pictures).

### Post-task ratings

After the scanning session, participants viewed the pictures again outside of the scanner in two blocks. In one block, participants rated the emotional intensity (‘How intense was your emotional response to this image?’) of all the pictures on a visual analog scale that stretched the width of the monitor (from 0 to 1024 pixels) anchored by ‘Low emotional intensity’ on the left, ‘Moderate emotional intensity’ in the middle and ‘High emotional intensity’ on the right. In a separate block, participants rated the self-relevance (‘How much did you personally associate with this image?’) of all the pictures on the same visual analog scale (except that the anchors specified low, moderate and high personal association). The self-relevance data are not presented here, but are available upon request. The order of the rating blocks was counterbalanced across participants.

### Behavioral analyses

#### Performance

To assess how well participants can maintain their emotional states, we calculated two metrics of performance (Mikels *et al.*, 2008; Gard *et al.*, 2011). For the idiographic agreement metric, we calculated the percentage of ‘maintain’ trials for which participants’ responses (e.g. higher) when comparing the emotional intensity of the two pictures matched the differences between the pictures in their post-task ratings (e.g. the second picture was rated as being more intense than the first). Because these ratings were *post hoc* and have been shown to be influenced by the act of maintaining the emotional state (C.E. Waugh and I.H. Gotlib, unpublished data), we also calculated a normative agreement metric in which we calculated the percentage of trials for which participants’ responses matched the normed ratings of the pictures.

#### Picture order bias

To assess participants’ differential responses to the first picture *vs* the second picture, which could reflect processes like decay (decreased endorsement of the first picture) or strategic enhancement (increased endorsement of the first picture), we calculated two simple metrics of bias. The first, ‘response bias’, was calculated as the percentage of maintenance trials for which participants chose the first picture as eliciting a more intense emotional response than the second picture. The second, ‘recall bias’, was calculated as the percentage of maintenance trials for which the participants’ post-task emotional intensity ratings were higher for the first picture of each pair than for the second picture.

#### Post-task ratings

To replicate our previous findings (C.E. Waugh and I.H. Gotlib, unpublished data) that maintaining emotion increases post-task ratings of the emotional intensity of both positive and negative pictures, we conducted a 2 (instructions: maintain, non-maintain) × 2 (valence: positive, negative) repeated measures analysis of variance on the post-task emotion intensity ratings of the first picture of each pair.

### fMRI data acquisition

BOLD data were acquired using a 3 T General Electric Magnetic Resonance scanner with a single channel, whole-head imaging coil. Head movement was minimized with padding around the participant’s head. BOLD data were then acquired with a single channel, whole-head imaging coil from 29 axial slices using a spiral pulse sequence (Glover and Law, 2001) [repetition time (TR) = 2000 ms, echo time (TE) = 40 ms, flip angle = 70°, field of view (FOV) = 22 cm, number of frames = 244]. Axial slices had 3.44 mm^2^ in-plane and 4 mm through-plane resolution (with 1 mm between-slice distance). A high-resolution structural scan (115 slices, 1 mm^2^ in-plane and 1.5 mm through-plane resolution, TE = min, flip angle = 15°, FOV = 22 cm) was performed following BOLD scanning runs.

### BOLD data analysis: preprocessing

Preprocessing was performed with the AFNI imaging analysis suite (Cox, 1996). BOLD images were slice-time corrected using the middle axial slice as the reference slice and then concatenated. Images were then motion corrected to the middle acquisition slice using Fourier interpolation. Data were then spatially smoothed with an 4 mm Gaussian kernel and warped to Talairach template space (voxel size = 3 mm^3^; Talairach and Tournoux, 1988). Next, multiple regression was used to remove nuisance effects including six head movement parameters (estimated from motion correction procedure), whole-brain global time series and linear drift. These images were then converted to statistical parametric mapping (SPM) analyze format, temporally smoothed (6 s kernel) and gray-matter masked with the participants’ averaged structural image.

### Functional analysis

We were interested in estimating BOLD activation due to processing the first picture as well as due to maintaining emotional information in the retention interval. To capture both neural responses to the first picture as well as responses that persisted into the retention interval, we used inverse logit (IL) modeling, a flexible HRF estimation procedure that can capture time-varying components of the BOLD response (Lindquist and Wager, 2007). HRFs derived from the IL modeling procedure have been shown to be better at estimating BOLD responses to emotional stimuli than is the canonical gamma HRF (Waugh *et al.*, 2010). SPM2 (Wellcome Department of Cognitive Neurology) was used, along with custom routines, to estimate the HRF to each picture type in each voxel. For the IL modeling approach, three parameters (height, time to inflection and slope of inflection) were estimated for each of the IL functions using a fast deterministic optimization algorithm that minimized sum of squares error. To balance flexibility with parameter interpretability, we chose the initial parameters (*V*_0_ = 3.94, 2.5, 0.47, 1.51, 4.27, 1.59, 5.14) to match the shape of a 16 TR (32 s) canonical gamma-based HRF, and the boundary limits of the parameters (*V*_lb:ub_ = 1.44:6.44, 1:5, 0:1.47, 0.51:2.51, 2.48:8.27, 0.59:2.59, 4.27:10.14) wide enough to flexibly capture time-varying components of the HRF, but narrow enough to avoid fitting the HRF to noise. The resulting IL functions were then summed to create the HRF from which the height (H), time-to-peak and width at half-height (W) of the BOLD responses were estimated.

We created contrasts for both height and width images to examine the effect of maintaining emotion *vs* not-maintaining emotion (maintain neg *vs* non-maintain neg; maintain pos *vs* non-maintain pos) and the general effect of viewing emotion images *vs* neutral images [only for non-maintenance trials, which were comparable to the neutral trials (non-maintain neg *vs* non-maintain neutral; non-maintain pos *vs* non-maintain neutral)]. For the group analysis, we used robust regression at the second level (Wager *et al.*, 2005), which minimizes the influence of outliers, to conduct random effects analyses on the contrasts. To balance controlling for Type I and Type II errors (Lieberman and Cunningham, 2009) as well as to capture smaller clusters, we implemented two statistical thresholds (Bennett *et al.*, 2009). Results for the contrasts were thresholded using a Monte Carlo simulation that calculates the cluster size needed for a per-voxel threshold of 0.001 to render a cluster-level corrected *P*-value of 0.05 (AlphaSim; Ward, 2000). For the more liberal threshold, we specified the Gaussian filter (fwhm) used in AlphaSim to account for spatial correlations among the voxels as equal to the spatial filter we applied during pre-processing (fwhm = 4), which accounts for applied, but not inherent spatial smoothing in the data. This procedure estimated a cluster size of *k* = 6 to obtain the corrected *P*-value of 0.05. For the conservative threshold, we estimated the spatial smoothing from the data (fwhm ∼ = 8, 8, 7.5), which led AlphaSim to estimate a cluster size of *k* = 17 to obtain the corrected *P*-value. In the tables, we note which threshold each cluster survived.

We next subjected the thresholded clusters in hypothesized regions and other regions of interest to independent follow-up selectivity analyses. First, we averaged the height and/or width estimates across all the voxels in the clusters of interest for both the contrasts on which these clusters were selected and the other contrasts. Then, we conducted *t*-tests on the values for the independent contrasts (as determined by the contrast independence equation presented in Kriegeskorte *et al.*, 2009 to avoid non-independent ‘double-dipping’) to avoid incorrectly claiming that a region was selectively active in contrast X, but not in an independent contrast Y. Finally, to analyze the correlation between behavior and brain activation, we computed Pearson correlation coefficients between the values from all of the contrasts of interest and the behavioral findings (which constitute independent statistical analyses because the clusters were not selected on the basis of the behavioral findings), and evaluated these correlations at a non-corrected threshold of *P* < 0.05.

## RESULTS

### Behavioral results

#### Performance

**Fig. 2:**
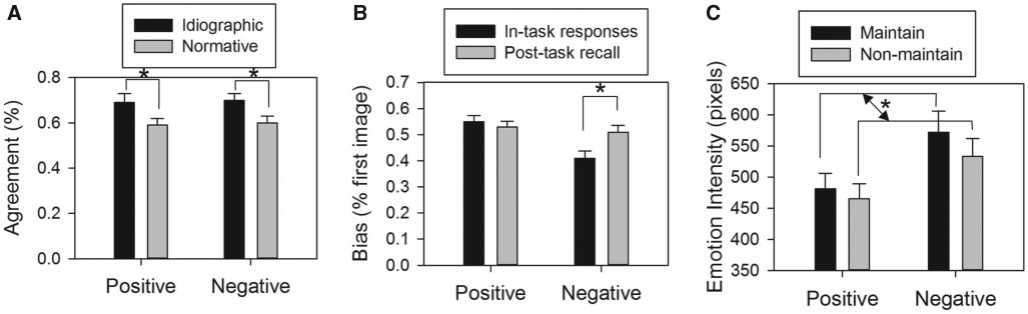
Behavioral findings. The graphs depict participants’ (A) idiographic and normative agreement: percentage of ‘maintain’ trials for which participants’ choice of which image was of higher intensity matched either their post-task ratings (idiographic) or normative ratings; (B) response and recall bias: percentage of ‘maintain’ trials for which participants’ either chose (in-task responses) or recalled (post-task recall) the first image as being more intense than the second image and (C) post-task ratings of the recalled emotion intensity of the images. *P < 0.05.

First, we examined participants’ idiographic and normative agreement of their choice of which picture in each pair elicited a more intense emotional response. There was no significant difference between idiographic agreement for pairs of negative pictures and agreement for pairs of positive pictures, *t*(23) = 0.23, *P* = 0.818, both of which were significant higher than chance (i.e. >0.50), *t*(23) = 6.45, *P* < 0.001, and *t*(23) = 4.82, *P* < 0.001, respectively (Figure 2A). There was also no significant difference between normative agreement for pairs of negative pictures and normative agreement for pairs of positive pictures, *t*(23) = 0.46, *P* = 0.649, both of which were significantly higher than chance, *t*(23) = 3.75, *P* < 0.001, and *t*(23) = 3.38, *P* = 0.003, respectively.

#### Bias

We next examined participants’ response bias toward the first picture of each pair. Participants were significantly less likely to choose the first negative picture as being more intense than the second negative picture in each pair, *t*(23) = 3.23, *P* = 0.004, whereas the reverse was true for the pairs of positive pictures, *t*(23) = 2.00, *P* = 0.057 (Figure 2B). These differential first-picture biases for positive and negative pictures were not evident in examining the recall bias toward each picture of the pair. Participants were equally likely to recall the first picture as being more intense than the second picture as they were the reverse for both negative picture pairs, *t*(23) = 0.21, *P* = 0.834, and positive picture pairs, *t*(23) = 1.45, *P* = 0.162 (Figure 2B).

This discrepancy between the response and recall bias for the negative pictures seemed to be due to the fact that during the task, participants were more likely to fail to choose the first negative picture of each pair as being more intense on the trials in which they recalled it as being more intense (mean = 0.39, s.e. = 0.046) than they were to fail to choose the second picture as being more intense on the trials in which they recalled it as being more intense (mean = 0.20, s.e. = 0.036), *t*(23) = 3.28, *P* = 0.003. For pairs of positive pictures, there were no significant differences between these two types of incongruities (means = 0.32, 0.26; s.e.s = 0.048, 0.043, respectively), *t*(23) = 1.16, *P* = 0.258.

#### Maintain vs non-maintain ratings

We compared participants’ post-task emotion intensity ratings of the first picture of each pair on the maintain trials to their ratings of the first picture on the non-maintain trials. This analysis yielded a significant main effect of valence, *F*(1,23) = 6.72, *P* = 0.016: participants recalled the negative pictures (mean = 553.04, s.e. = 30.60) as being more emotionally intense than they did the positive pictures (mean = 473.18, s.e. = 23.59; Figure 2C). Replicating previous findings, the analysis also yielded a main effect of instructions, *F*(1,23) = 6.72, *P* = 0.016: participants remembered the pictures on the ‘maintain’ trials as being more emotionally intense (mean = 526.74, s.e. = 23.86) than they did the pictures on the ‘non-maintain’ trials (mean = 499.47, s.e. = 22.35; Figure 2C). The interaction of valence and instructions was not significant, *F*(1,23) = 1.36, *P* = 0.255.

#### Summary

The performance findings indicate that participants were able to maintain enough emotional information to perform above chance in this task. These findings were equivocal, however, in supporting either the active or simple emotional maintenance hypotheses. On one hand, the finding that participants tended to select the first picture as being less negative than the second picture suggests that the intensity of the first picture decayed over the maintenance period, consistent with the simple maintenance hypothesis. Alternatively, however, after the task, participants recalled these first pictures as being equally intense as the second picture of each pair and more intense than the first pictures on the non-maintain trials, which suggests that participants’ elaboration of their emotional state to these pictures increased the recalled intensity of these states at the end of the task.

### fMRI results

#### Maintain vs non-maintain emotion

**Fig. 3:**
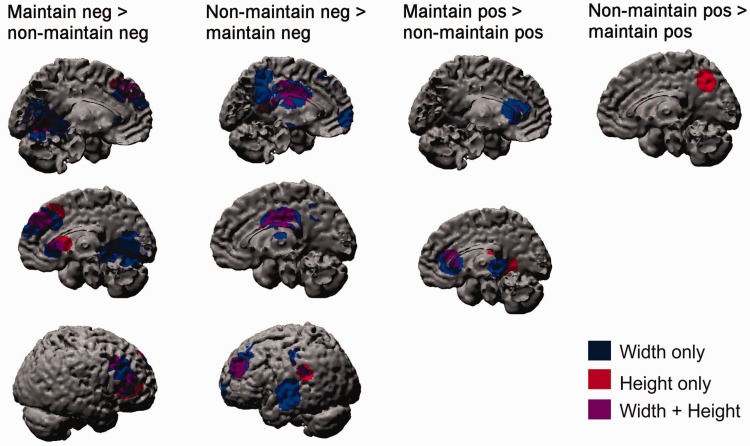
Brain regions that exhibited differential height and/or width of BOLD activation in the maintain *vs* non-maintain negative and positive emotion contrasts.

**Fig. 4:**
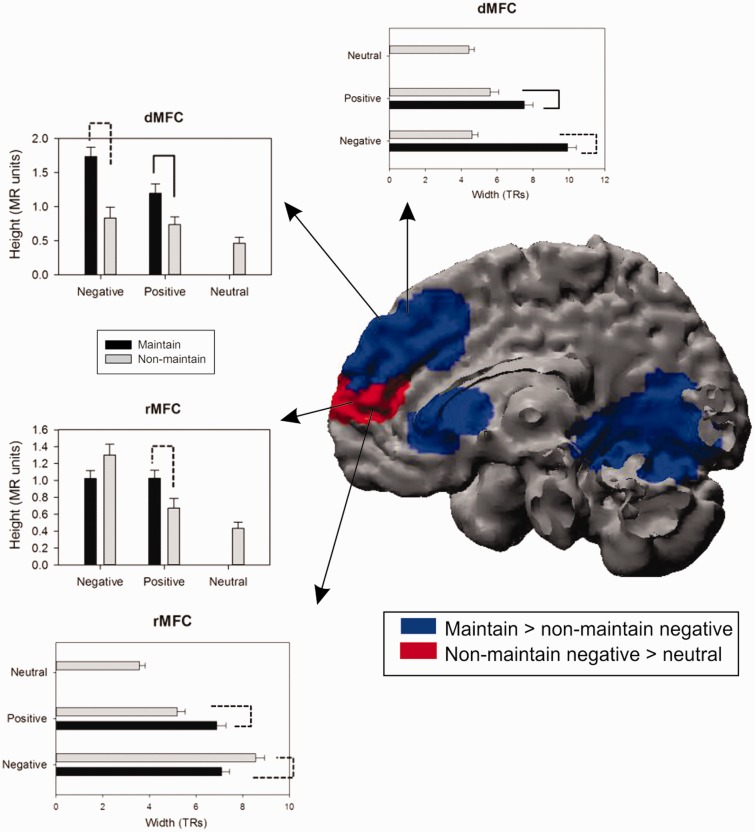
Height and width of BOLD activation in the dorsal and rMFC. Regions in blue exhibited greater height and/or width of BOLD activation in the maintain *vs* non-maintain negative contrast. Regions in red exhibited greater height and/or width of BOLD activation in the non-maintain negative *vs* neutral contrast. Solid bracket lines indicate that *P* < 0.05 for independent contrast *t*-tests. Dotted bracket lines indicate that *P* < 0.05 for non-independent contrast *t*-tests (i.e. the data being analyzed were already selected for exhibiting a strong effect), so caution should be exercised when making inferences.

We tested whether ‘active’ or ‘passive’ maintenance better characterizes how the brain maintains emotional states. Supporting the ‘active maintenance’ hypothesis, the regions that exhibited greater height and/or width of activation when participants maintained negative emotion than when they did not maintain negative emotion included the dMFC (4, 40, 38; Figure 3), right dlPFC (46, 32, 10 and 40, 16, 34) and right caudate (14, 22, 4; Table 1; Figure 3), which is also implicated in active maintenance of information (Chang *et al.*, 2007). An overlapping region in the caudate also exhibited greater activation when participants were maintaining *vs* not maintaining positive emotion (8, 26, 10; Table 2; Figure 3). Follow-up independent *t*-tests indicated that the dMFC also showed greater activation in this contrast (Figure 4). Although most of these regions exhibited both greater height and width of activation, cluster sizes were consistently larger for the width estimates, most notably in the occipital cortex (345 voxels *vs* 7 voxels; Table 1) for the maintain *vs* not-maintain negative emotion contrast. This observation supports our assessment of the duration of BOLD activation as a valid index of the maintenance of emotion.

**Table 1:** Regions shown by the IL model to have significantly different height and/or width of activation among the maintain negative, non-maintain negative and non-maintain neutral conditions

Region	*x*	*y*	*z*	Voxels	Volume	Peak *z*
Height						
Maintain neg > non-maintain neg						
r. superior temporal G.	52	−22	−8	7	189	5.14
Lingual G.	−8	−68	−2	7	189	5.37
r. caudate	14	22	4	8	216	3.97
r. middle/inferior frontal G.	46	32	10	26^a^	702	5.35
r. superior frontal G.	40	16	34	16	432	5.12
Dorsal medial frontal G.	4	40	38	12	324	6.13
r. precentral G.	10	26	46	9	243	4.63
Non-maintain neg > maintain neg						
Posterior cingulate	−14	−44	20	11	297	4.91
l. postcentral G.	−44	−38	34	69^a^	1863	6.06
Middle cingulate	−2	−10	28	47^a^	1269	6.60
l. middle frontal G.	−26	44	28	26^a^	702	5.49
Non-maintain neg > non-maintain neut						
l. superior temporal G.	−50	−16	−4	12	324	4.98
r. superior temporal G.	50	−32	14	7	189	4.62
Rostral medial frontal G.	10	52	16	20^a^	540	4.54
Rostral medial frontal G.	−4	64	14	7	189	4.64
r. postcentral G.	52	−32	22	7	189	5.43
l. postcentral G.	−44	−34	34	6	162	4.50
Non-maintain neut > non-maintain neg						
r. precentral G.	38	−2	28	6	162	4.33
Width						
Maintain neg > non-maintain neg						
Lingual G.	−4	−62	−2	345^a^	9315	37.25
r. caudate	10	26	2	9	243	5.14
r. middle/inferior frontal G.	46	32	16	13	351	4.94
r. middle frontal G.	44	22	26	11	297	4.93
Dorsal medial frontal G.	4	44	34	32^a^	864	8.96
r. superior frontal G.	44	16	34	9	243	4.81
Non-maintain neg > maintain neg						
l. superior temporal G.	−58	−14	2	27^a^	729	5.97
Rostral medial frontal G.	−10	62	−2	9	243	4.86
Posterior cingulate	−10	−46	26	24^a^	648	6.53
Middle cingulate	−2	−10	28	112^a^	3024	7.18
l. middle frontal G.	−28	44	28	32^a^	864	6.00
l. postcentral G.	−44	−34	34	86^a^	2322	7.71
l. middle frontal G.	−28	32	34	7	189	5.89
l. superior frontal G.	−16	26	46	6	162	4.32
Precuneus	−8	−50	50	7	189	4.36
Non-maintain neg > non-maintain neut						
l. superior temporal G.	−46	−20	−2	8	216	4.13
Rostral medial frontal G.	−14	62	2	10	270	5.96
Rostral medial frontal G.	−4	64	14	13	351	4.89
Rostral medial frontal G.	8	52	16	41^a^	1107	5.97
Rostral medial frontal G.	−14	64	22	9	243	5.09
r. insula/supramarginal G.	52	−32	22	10	270	6.42
Rostral/dorsal medial frontal G.	−4	56	28	6	162	4.85
Non-maintain neut > non-maintain neg						
r. hippocampus	22	−22	−20	7	189	4.79
l. hippocampus	−26	−34	−16	6	162	4.12
Cuneus	−4	−70	4	7	189	3.79
Cuneus	−20	−74	16	13	351	4.26

Neg, negative; pos, positive; neut, neutral; r., right; l., left, G., gyrus.

^a^Indicates that the cluster survived the more conservative threshold.

**Table 2:** Regions shown by the IL model to have significantly different height and/or width of activation among the maintain positive, non-maintain positive and non-maintain neutral conditions

Region	*x*	*y*	*z*	Voxels	Volume	Peak *z*
Height						
Maintain pos > non-maintain pos						
r. parahippocampal G.	22	−44	4	15	405	5.11
r. thalamus	26	−28	8	8	216	4.18
r. caudate	8	26	10	6	162	5.49
Non-maintain pos > maintain pos						
No clusters meet threshold						
Non-maintain pos > non-maintain neut						
r. superior temporal G.	52	−26	−2	12	324	5.55
Non-maintain neut > non-maintain pos						
No clusters meet threshold						
Width						
Maintain pos > non-maintain pos						
r. thalamus	10	−32	2	6	162	4.63
r. caudate	8	28	8	19^a^	513	5.20
Anterior cingulate	−8	22	14	6	162	5.16
Non-maintain pos > maintain pos						
Precuneus	8	−58	40	7	189	6.36
Non-maintain pos > non-maintain neut						
r. superior temporal G.	50	−26	−2	7	189	4.25
Non-maintain neut > non-maintain pos						
l. hippocampus	−20	−26	−16	8	216	4.56
Thalamus	−2	−26	−4	6	162	6.77

Neg, negative; pos, positive; neut, neutral; r., right; l., left, G., gyrus.

^a^Indicates that the cluster survived the more conservative threshold.

Notably, there were clusters of activation that showed the reverse pattern—greater height/width of activation when participants were not maintaining *vs* maintaining emotion. Specifically, the middle/posterior cingulate cortex (−2, 10, 28 and −10, −46, 26) exhibited this pattern for negative emotion (Figure 4), and the precuneus (8, −58, 40) exhibited this pattern for positive emotion.

#### Non-maintain emotion vs non-maintain neutral

Consistent with previous research, participants exhibited greater height and width of rMFC activation (e.g. 8, 52, 16) when viewing the non-maintained negative emotion pictures than when viewing the non-maintained neutral pictures (Table 1; Figure 3). Again there were more active voxels in this region that exhibited greater width (73) than greater height (27), which is consistent with our previous finding that the duration of rMFC activation is more tightly associated with processing intense emotional stimuli than is its height (Waugh *et al.*, 2010). Other regions in this contrast included the left superior temporal gyrus (−50, −16, −4) and right insula/supramarginal gyrus (52, −32, 22). Only the right superior temporal gyrus (50, −26, −2) exhibited greater activation to non-maintain positive emotion images than to non-maintain neutral images.

#### Correlations between brain and behavior

**Fig. 5:**
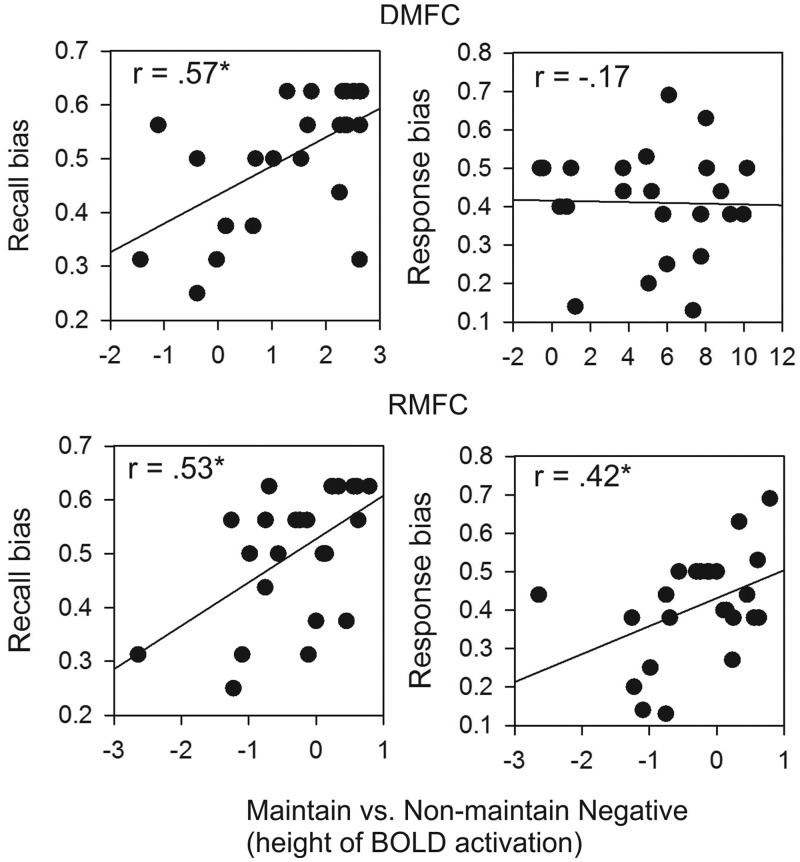
Scatterplots of the correlations between behavior (recall and response bias) and dorsal and rMFC height of activation in the maintain *vs* non-maintain negative contrast. *P < 0.05.

The behavioral findings indicated that participants exhibited a slight decay in their in-the-moment maintenance of emotion (decreased response bias), but may have elaborated on their emotional intensity when maintaining emotion to try to overcome this decay (increased recall bias). We next examined which brain regions were associated with these processes, and with performance in general, by correlating activation in the dMFC (4, 44, 34), rMFC (8, 52, 16) and other regions of interest with response and recall bias toward the first picture, and with idiographic agreement. Differential activation in the dMFC and dlPFC when maintaining *vs* not maintaining negative emotion was significantly positively correlated with biased recall of the first negative picture of each pair, but not with biased response (*r*s < |0.17|) or with agreement (*r*s < |0.21|; Figure 5). Recall bias was positively associated with dMFC activation height, *r* = 0.57, *P* = 0.004, and width (4, 44, 34), *r* = 0.52, *P* = 0.01, as well as with activation height in lateral PFC regions, including the right middle/inferior frontal gyrus (46, 32, 10), *r* = 0.47, *P* = 0.02, and precentral gyrus (10, 26, 46), *r* = 0.43, *P* = 0.038.

Both height and width of activation in the rMFC (Figure 4) when maintaining *vs* not maintaining negative emotion were positively correlated with recall bias, *r*s = 0.53 and 0.41, *P*s = 0.007, 0.047, respectively (Figure 5). This pattern is similar to that found in the dMFC, but offers additional predictive power above and beyond that of dMFC. Activation in both the dMFC (height) and the rMFC (height) predicted recall bias when controlling for activation in the other region, βs = 0.44, 0.38, *P*s = 0.018, 0.036, respectively. Unlike the dMFC, however, activation in the rMFC was also positively correlated with response bias (height), *r* = 0.42, *P* = 0.042. Also unlike the dMFC, differential width of activation in the rMFC when participants were maintaining *vs* not maintaining positive emotion was positively correlated with recall bias, *r* = 0.41, *P* = 0.045.

Idiographic agreement was not correlated with dMFC or any lateral PFC regions. Instead, in the maintain *vs* non-maintain negative emotion contrast, agreement was correlated positively with caudate activation width (14, 22, 4), *r* = 0.53, *P* = 0.008, and negatively with superior temporal gyrus activation height (52, −22, −8), *r* = −0.41, *P* = 0.047. In the maintain *vs* non-maintain positive emotion contrast, several regions were negatively correlated with agreement, including rMFC width, *r* = −0.44, *P* = 0.03, caudate activation width (8, 28, 8), *r* = −0.47, *P* = 0.02,and precuneus activation width (8, −58, 40), *r* = −0.44, *P* = 0.033.

## DISCUSSION

In this study, we investigated the neural underpinnings of emotional state maintenance. The findings generally support the ‘active’ maintenance of emotion hypothesis by showing that intentionally maintaining emotional states to compare them with subsequent emotional states activated the dMFC and dlPFC more so than not intentionally maintaining emotional states. The dMFC was correlated with recalling the first negative image of each pair as more emotionally intense than the second image, but was not correlated with in-the-moment biased selection of the first negative image as more intense. This suggests that the dMFC was involved in the elaboration of the emotional intensity of the images, which caused this information to be encoded more deeply and, therefore, more easily retrieved later (Craik and Tulving, 1975), but that this elaboration was in the service of maintaining the emotional state in the moment rather than up-regulating it. In addition, the dMFC was associated with both negative and positive (albeit to a lesser degree) maintenance, suggesting that it is a valence-general mechanism.

With the dMFC, the dlPFC was also more active when maintaining emotion than when not maintaining emotion. The dlFPC is part of the central executive that is involved, among other functions, with manipulating contents in working memory (Smith and Jonides, 1999). This provides further evidence that participants were actively maintaining their emotional states. The differentiation of the functions between the dMFC and the dlPFC, however, is less clear. One possibility is that the dMFC was responsible for generating the maintained emotional state by elaborating on the existing emotional state, whereas the dlPFC was responsible for the manipulation of that information in working memory to compare it appropriately to the subsequent emotional state. This formulation is consistent with the behavioral evidence presented earlier for the dMFC and recall bias and also with the caudate findings. The dlPFC has extensive connections with the anterior caudate (Postuma and Dagher, 2006), which was also more active when participants were maintaining emotion than when they were not maintaining emotion. The caudate has been posited to be responsible for the adaptive facilitation of working memory—knowing when to maintain and when to update (O'Reilly and Frank, 2006), which is consistent with the findings from this study that caudate activation was correlated with task performance.

The current neural and behavioral findings, along with previous behavioral findings (Mikels *et al.*, 2008; C.E. Waugh and I.H. Gotlib, unpublished data) clearly support the formulation that people can actively maintain their emotional states when instructed to do so. This active maintenance of emotion, however, is not the only mechanism by which emotions endure past the end of the emotion-eliciting stimulus. Evidence from previous fMRI studies (Waugh *et al.*, 2010) and this study suggest that the rMFC is involved with the ‘passive’ maintenance of emotion, i.e. the implicit or uninstructed generation and perpetuation of emotional states. Participants exhibited greater height and duration of activation in this region when they viewed negative emotion pictures than when they viewed neutral pictures, suggesting that the rMFC was associated with the initial generation and continuation of the emotional state. More telling, however, is that relatively less activation in the rMFC when instructed to maintain (*vs* non-maintain) negative emotion was correlated with a tendency for participants to endorse the first picture of each pair as less intense than the second picture. This pattern of results suggests that the rMFC tracked the natural duration of the emotional states generated by the first picture, but that these states decayed slightly before the presentation of the second picture.

The amygdala and insula did not seem to play role in the maintenance of emotion. Although a small region in the posterior insula exhibited greater width of activation when participants were viewing negative emotion pictures than when they were viewing neutral pictures, there was not a significant correlation between activation in this region and any of the behavioral metrics; it is unclear, therefore, whether the insula was involved in the passive maintenance of emotion. It is possible that we did not find amygdala activation in any of our contrasts because we used complex emotional scenes; investigators have found amygdala activation to be weaker in response to complex scenes than to other forms of emotional stimuli (especially faces; Hariri *et al.*, 2002). Future investigations that use emotional faces instead of complex scenes may elucidate whether the amygdala is indeed involved in the maintenance of emotional states.

Previous studies using this emotional maintenance paradigm have shown that people can successfully maintain their emotional state despite intervening cognitive distractors during the delay period (Mikels *et al.*, 2008), suggesting that the maintenance of emotional states and of cognitive information are at least partly dissociable. Further supporting the independence of cognitive and emotional state working memory, investigators have found that individuals with severe anterograde amnesia were able to maintain emotional states beyond their episodic memory of the eliciting stimulus (Feinstein *et al.*, 2010), and that psychopathological deficits in emotional working memory were not due to deficits in other non-emotion forms of working memory (Gard *et al.*, 2011). This evidence suggests that participants in this study were recruiting their dMFC to maintain their emotional state rather than their cognitive representations of their emotional state (e.g. as an episodic memory or a numerical value). It is possible, however, that the dMFC and dlPFC were also recruited to maintain task goals, which differed between the maintain and non-maintain conditions. To test these formulations, fMRI investigations of emotional maintenance should place cognitive distractors in the intervening delay period and control for task goal maintenance.

These data add to the evidence that using flexible time-varying HRFs is useful for understanding the temporal dynamics of emotion. By definition, the maintenance of emotion is a temporal process that increases the duration of emotion. We used time-varying HRFs that could capture this duration of emotion by estimating the width of BOLD activation. And although there were large overlaps in the clusters that exhibited differences in activation height and the clusters that exhibited differences in activation width, there was also notable non-overlap. For example, in the maintain *vs* non-maintain negative emotion contrast, there were almost 50 times as many voxels in the lingual gyrus and 3 time as many voxels in the middle cingulate that were more sensitive to differences in activation width than to differences in activation height. Future research is needed, however, to allow us to draw inferences about the psychological meaning of these differences in width of emotion-related BOLD activation.

In sum, these data provide evidence for both the active and passive models of emotion maintenance. Whereas the rMFC is involved in the implicit generation and duration of emotional responding, the dMFC is involved in the intentional elaboration and maintenance of that state. This neural model of how emotions may endure past the duration of the stimuli that elicited them can inform the emotion regulation literature, especially those studies investigating the irregularities in the duration of emotional experiences found in multiple forms of psychopathology (Gilboa and Gotlib, 1997; Young *et al.*, 2000; Gard *et al.*, 2011). For example, this study presents the possibility that the emotion maintenance deficits found in persons with schizophrenia may be related to the abnormal structure of their MFC (Pomarol-Clotet *et al.*, 2010) and/or connectivity of their MFC with other regions (Whitfield-Gabrieli *et al.*, 2009). This study also supports the use of the rMFC as a target region for examining the prolongation of negative emotion exhibited by people with depression (Waugh *et al.*, 2012), and goes further to suggest that the dMFC plays a role in the strategic maintenance of emotion-related thoughts seen in certain forms of rumination (Nolen-Hoeksema and Morrow, 1993), worry (Borkovec *et al.*, 1998) and other perseverative cognitions (Brosschot *et al.*, 2006). These formulations are conjectural and should be considered with caution, especially because our task used very simple, brief stimuli and the enduring emotions that characterize various forms of psychopathology are typically much longer. These formulations, however, do highlight the many areas of inquiry that may benefit from the present data that increase our understanding of the neural mechanisms that underlie emotion maintenance.

## SUPPLEMENTARY DATA

Supplementary data are available at *SCAN* online.

Supplementary Data
