# Supplementary material for: The role of the medial frontal cortex in the maintenance of emotional states
Source: Soc Cogn Affect Neurosci. 2014 Mar 10;9(12):2001–9. doi: 10.1093/scan/nsu011 (PMC4249480; doi:10.1093/scan/nsu011)
Supplement: Supplementary Data [file supp_9_12_2001__index.html]

The Role of the Medial Frontal Cortex in the Maintenance of Emotional States — The role of the medial frontal cortex in the maintenance of emotional states — The role of the medial frontal cortex in the maintenance of emotional states — Supplementary Data 

# The role of the medial frontal cortex in the maintenance of emotional states

## Supplementary Data

files

**Files in this Data Supplement:**

- Supplementary Data - docx file
