# Supplementary material for: The role of the medial frontal cortex in the maintenance of emotional states
Source: Soc Cogn Affect Neurosci. 2014 Mar 10;9(12):2001–9. doi: 10.1093/scan/nsu011 (PMC4249480; doi:10.1093/scan/nsu011)
Supplement: Supplementary Data [file supp_nsu011_scan-13-236-File007.docx]

Supplementary Material for Waugh, Lemus, & Gotlib “The role of medial frontal cortex in the maintenance of emotional states”

Methods

Stimulus information

We selected the stimuli (pictures) for this study by first omitting IAPS erotic pictures and then equating the four emotional trial types on established normed ratings ([Lang, Bradley, & Cuthbert, 1997](#_ENREF_16)) of intensity of emotional valence (intensity for positive pictures was reverse-coded for comparability): maintain-positive = 3.65, non-maintain-positive = 3.68, maintain-negative = 3.64, non-maintain-negative = 3.60, F(1,76) = .15, p = .699. We then included less intense neutral pictures: non-maintain-neutral = 4.22, F(1,98) = 50.23, p < .001 (vs. four other conditions). On half of the trials within each of the emotion conditions the second image was normed as more intense (M = 4.12, SD = .47) than the first image (M = 3.16, SD = .43), t(39) = 14.86, p < .001; on the other half of the trials, the second image was normed as less intense (M = 3.13, SD = .41) than the first image (M = 4.16, SD = .43), t(39) = 12.37, p < .001. Assignment of the individual pictures to each condition was not counterbalanced across participants.

Table 1

List of image names used in the emotional maintenance task

| Maintain – Negative | Non-maintain – Negative | Maintain – Positive | Non-maintain – Positive | Non-maintain - Neutral |
| --- | --- | --- | --- | --- |
| 1220.bmp | 6260.bmp | 5629.bmp | 8460.bmp | 60323.bmp |
| 9101.bmp | 5971.bmp | 2560.bmp | 2170.bmp | 2385.bmp |
| 1111.bmp | 6210.bmp | 60288.bmp | 8130.bmp | 2580.bmp |
| 7380.bmp | 3300.bmp | 60084.bmp | 1604.bmp | 7705.bmp |
| 9620.bmp | 6213.bmp | 1340.bmp | 5991.bmp | 5532.bmp |
| 1302.bmp | 9830.bmp | 8116.bmp | 2070.bmp | 2681.bmp |
| 3160.bmp | 1030.bmp | 1460.bmp | 5621.bmp | 7160.bmp |
| 3220.bmp | 9622.bmp | 60112.bmp | 60316.bmp | 93073.bmp |
| 3210.bmp | 2141.bmp | 1811.bmp | 2050.bmp | 9070.bmp |
| 1390.bmp | 3500.bmp | 1500.bmp | 1710.bmp | 5000.bmp |
| 9110.bmp | 9373.bmp | 1590.bmp | 7195.bmp | 9700.bmp |
| 9160.bmp | 5940.bmp | 60267.bmp | 8490.bmp | 4610.bmp |
| 6370.bmp | 9584.bmp | 60224.bmp | 8030.bmp | 2620.bmp |
| 2120.bmp | 1080.bmp | 60262.bmp | 8503.bmp | 5130.bmp |
| 9480.bmp | 1230.bmp | 2540.bmp | 1610.bmp | 2810.bmp |
| 9561.bmp | 8230.bmp | 2209.bmp | 1660.bmp | 7190.bmp |
| 9571.bmp | 3230.bmp | 5910.bmp | 1650.bmp | 2514.bmp |
| 1274.bmp | 3280.bmp | 93060.bmp | 8185.bmp | 2383.bmp |
| 2692.bmp | 2810.bmp | 8501.bmp | 8034.bmp | 2220.bmp |
| 1945.bmp | 1110.bmp | 5982.bmp | 8041.bmp | 2130.bmp |
| 9180.bmp | 6010.bmp | 60018.bmp | 8300.bmp | 7620.bmp |
| 6360.bmp | 2682.bmp | 2311.bmp | 8170.bmp | 93025.bmp |
| 1270.bmp | 6211.bmp | 60152.bmp | 2040.bmp | 93079.bmp |
| 9300.bmp | 2700.bmp | 5890.bmp | 2057.bmp | 7283.bmp |
| 1090.bmp | 6200.bmp | 60273.bmp | 5950.bmp | 1670.bmp |
| 1052.bmp | 7360.bmp | 8220.bmp | 5830.bmp | 7140.bmp |
| 1070.bmp | 9594.bmp | 60220.bmp | 8497.bmp | 7090.bmp |
| 9050.bmp | 9404.bmp | 5594.bmp | 1721.bmp | 7920.bmp |
| 6410.bmp | 2271.bmp | 8600.bmp | 93052.bmp | 7830.bmp |
| 1019.bmp | 9440.bmp | 2550.bmp | 1540.bmp | 5410.bmp |
| 6840.bmp | 3250.bmp | 8280.bmp | 1810.bmp | 93049.bmp |
| 6930.bmp | 3022.bmp | 60134.bmp | 2345.bmp | 2487.bmp |
| 8231.bmp | 1051.bmp | 2240.bmp | 60025.bmp | 7620.bmp |
| 9230.bmp | 9560.bmp | 1850.bmp | 60099.bmp | 6150.bmp |
| 9280.bmp | 9181.bmp | 5201.bmp | 1440.bmp | 6150.bmp |
| 2490.bmp | 9290.bmp | 8250.bmp | 4601.bmp | 5220.bmp |
| 1201.bmp | 9331.bmp | 1942.bmp | 8180.bmp | 1313.bmp |
| 9582.bmp | 1113.bmp | 2260.bmp | 60204.bmp | 7490.bmp |
| 6300.bmp | 1300.bmp | 4599.bmp | 8117.bmp | 5533.bmp |
| 7361.bmp | 5972.bmp | 4598.bmp | 5831.bmp | 7402.bmp |
